# 220D-F2 from *Rubus ulmifolius* Kills *Streptococcus pneumoniae* Planktonic Cells and Pneumococcal Biofilms

**DOI:** 10.1371/journal.pone.0097314

**Published:** 2014-05-13

**Authors:** Sharmila J. Talekar, Sopio Chochua, Katie Nelson, Keith P. Klugman, Cassandra L. Quave, Jorge E. Vidal

**Affiliations:** 1 Hubert Department of Global Health, Rollins School of Public Health, Atlanta, Georgia, United States of America; 2 Center for the Study of Human Health, Emory College of Arts and Sciences, Atlanta, Georgia, United States of America; 3 Department of Dermatology, School of Medicine, Emory University, Atlanta, Georgia, United States of America; The Scripps Research Institute and Sorrento Therapeutics, Inc., United States of America

## Abstract

*Streptococcus pneumoniae* (pneumococcus) forms organized biofilms to persist in the human nasopharynx. This persistence allows the pneumococcus to produce severe diseases such as pneumonia, otitis media, bacteremia and meningitis that kill nearly a million children every year. While bacteremia and meningitis are mediated by planktonic pneumococci, biofilm structures are present during pneumonia and otitis media. The global emergence of *S. pneumoniae* strains resistant to most commonly prescribed antibiotics warrants further discovery of alternative therapeutics. The present study assessed the antimicrobial potential of a plant extract, 220D-F2, rich in ellagic acid, and ellagic acid derivatives, against *S. pneumoniae* planktonic cells and biofilm structures. Our studies first demonstrate that, when inoculated together with planktonic cultures, 220D-F2 inhibited the formation of pneumococcal biofilms in a dose-dependent manner. As measured by bacterial counts and a LIVE/DEAD bacterial viability assay, 100 and 200 µg/ml of 220D-F2 had significant bactericidal activity against pneumococcal planktonic cultures as early as 3 h post-inoculation. Quantitative MIC’s, whether quantified by qPCR or dilution and plating, showed that 80 µg/ml of 220D-F2 completely eradicated overnight cultures of planktonic pneumococci, including antibiotic resistant strains. When preformed pneumococcal biofilms were challenged with 220D-F2, it significantly reduced the population of biofilms 3 h post-inoculation. Minimum biofilm inhibitory concentration (MBIC)_50_ was obtained incubating biofilms with 100 µg/ml of 220D-F2 for 3 h and 6 h of incubation. 220D-F2 also significantly reduced the population of pneumococcal biofilms formed on human pharyngeal cells. Our results demonstrate potential therapeutic applications of 220D-F2 to both kill planktonic pneumococcal cells and disrupt pneumococcal biofilms.

## Introduction

*Streptococcus pneumoniae* (pneumococcus) is an important human pathogen associated with high morbidity and mortality [1]. The global burden of pneumococcal infection is especially high in children, immunocompromised patients and the elderly causing severe illnesses such as pneumonia, otitis media, bacteremia and meningitis [2]–[4]. The pneumococcus causes 15 million cases of serious diseases [1], [5] and 1.6 million deaths each year including ∼1 million child deaths in those under 5 years of age [6]. In the US, the pneumococcus is responsible for more than 6 million cases of otitis media, ∼500,000 cases of pneumonia, ∼50,000 cases of bacteremia, and ∼3,000 cases of meningitis. Only in the elderly, the annual cost of pneumococcal diseases, to the US government, is nearly $5.5 billion [7].

Colonization of the human nasopharynx, which occurs in early childhood, and persistence in this niche are prerequisites for pneumococcal disease [4], [8]. The pneumococcus persists for months in the nasopharynx forming specialized structures referred to as biofilms [9], [10]. Biofilms are structured communities of bacterial cells enclosed in a self-produced polymeric matrix composed of polysaccharides, proteins and nucleic acids that is adherent to inert or living surfaces [11]. In comparison to their planktonic counterparts (10^8^ CFU/ml), biofilm bacteria reach a much higher density (10^11^ CFU/ml) which may impact the pharmacodynamics of antibiotics [12]. Pneumococci embedded in these biofilms can migrate to other anatomic sites to cause severe biofilm-associated diseases such as pneumonia, and otitis media, [13]–[15]. From the lungs of patients with pneumococcal pneumonia or the ear cavity when causing otitis media, planktonic pneumococci can disperse from the biofilm structure and invade sterile sites such as the blood stream or brain, to cause lethal bacteremia or meningitis, respectively [16]–[18].

It has been estimated that ∼60% of bacterial infections and up to 80% of chronic infections are mediated by organisms producing biofilms [19]–[21]. Besides pneumococcal pneumonia and otitis media, other infectious diseases in which biofilms have been implicated include dental plaque, gingivitis, endocarditis, musculoskeletal infections, and urinary tract infections [22], chronic wounds [23], coating contact lenses [24]. Biofilm-related infections lead to longer hospital stays, recurrent infections, and increased fatalities in the most recalcitrant infections [19], [25]–[30]. Moreover, biofilm-associated cells are between 10^2^ to 10^3^ fold less susceptible to antibiotics than their planktonic counterparts [26], [31]–[33].

With the emergence of multi-drug resistance clones there is increase in antibiotic resistance in *S. pneumoniae* strains [34]. Whereas intrinsic mechanisms of antibiotic resistance of planktonic pneumococci have been thoroughly studied [35], [36], the specific mechanism by which pneumococcal biofilms increase antibiotic resistance is under active investigation. A study by Garcia-Castillo *et al* (2007) demonstrates that biofilms formed by *S. pneumoniae* strains, isolated from cystic fibrosis patients, had a significantly higher resistance to penicillin, tetracycline and rifampicin than did the same strains grown as planktonic pneumococci [37]. Increased resistance of biofilm cells, in comparison to planktonic cultures, have also been reported for amoxicillin, erythromycin, clindamycin and levofloxacin [38], and for cefazolin and vancomycin [39]. More recent *in vivo* studies by Marks *et al* (2012), using a mouse model of persistence, showed that pneumococcal biofilms formed on nasopharyngeal tissue were more resistant to gentamicin and penicillin G than did pneumococci dispersed from the biofilm structure [40].

Natural products and related structures are increasingly becoming essential sources of new pharmaceuticals, because of the immense variety of functionally relevant secondary metabolites. Our previous studies discovered that an extract from the plant *Rubus ulmifolius* Schott., Rosaceae (Elmleaf blackberry) showed antimicrobial activity (ranging from 50–200 µg/ml of crude extract) against *Staphylococcus aureus* strains with no damage to human mammalian cells [41]. Sophisticated studies using LC-UV/MS/MS (liquid chromatography-ultraviolet absorption tandem mass spectrometry) revealed that the extract, hereafter referred as 220D-F2, contained ellagic acid (EA) and several ellagic acid derivatives (EADs) or sapogenin-related compounds. 220D-F2 was able to limit formation of biofilms made by methicillin-resistant *S. aureus* (MRSA) strains belonging to different linages, (i.e. USA100, USA200, etc.). Tested strains included community-acquired, global epidemic and MRSA strain USA300 [41]. MRSA strains bear resistance to all penicillins and other β–lactam antimicrobial drugs and produce severe human diseases such as skin and soft tissue infections (SSTIs), endovascular infections, pneumonia, septic arthritis, endocarditis, osteomyelitis, foreign-body infections, and sepsis [41]–[43]. Our previous experiments also demonstrate therapeutic potential of 220D-F2 since it decreased staphylococcal biofilm counts formed on catheters *in vitro*, and offered a more significant reduction of biofilm counts when incubated along with antibiotics such as daptomycin or clindamycin [41].

The aim of the present study was to assess the antibacterial effect of 220D-F2 against planktonic pneumococci and pneumococcal early and mature biofilms. We demonstrate here that 220D-F2 kills planktonic pneumococci and therefore limits the formation of biofilm structures. 220D-F2 was also able to eradicate early and mature biofilms and biofilms made on human pharyngeal cells. Together, our studies highlight the potential prophylactic and therapeutic applications of 220D-F2 against pneumococcal diseases involving either planktonic cells (i.e. bacteremia and meningitis) or biofilm-related diseases such as otitis media and pneumonia.

## Materials and Methods

### Bacterial Strain and Culture Conditions

Strains used in this study were reference genome-sequenced *S. pneumoniae* strain D39 [44] (GenBank accession # NC_008533), TIGR4 [45] and SPJV01. D39 is a virulent encapsulated type 2 strain [46], TIGR4 is another invasive isolate that belongs to vaccine serotype 4 and SPJV01 a D39-derivative expressing the green fluorescent protein (GFP) [47]. *S. pneumoniae* strain GA58771 is resistant to amoxicillin, cefuroxime, clindamycin, erythromycin, penicillin and tetracycline. Strains TN33388 [48] and 7828–04 are resistant to chloramphenicol, erythromycin, and intermediate resistant to linezolid [49]. *S. pneumoniae* strains were cultured on Trypticase Soy Agar plates with 5% Sheep Blood (BAP), Muller-Hinton (MH) broth, or Todd-Hewitt broth containing 0.5% (wt/vol) of yeast extract (THY).

### Preparation of Extract 220D-F2

220D-F2 was prepared as previously described [41]. Briefly, voucher specimens of the plant were deposited at the Emory University Herbarium (GEO) and bulk samples of the roots were dried, ground into a fine powder and extracted in 95% ethanol. The plant material was removed via vacuum filtration and the solvent removed through rotary evaporation and lyophilization. The resulting extract was resuspended in water and subjected to successive partitioning in hexanes, ethyl acetate, and butanol using a separatory funnel. The butanol partition was dried down, and then further separated using a gravity column with a methanol and dichloromethane gradient (the active fraction, 220D-F2 being collected at 40∶60 MeOH:CH_2_Cl_2_).

### 220D-F2 Quality Control Testing

All batches of 220D-F2 were subjected to high-performance liquid chromatography (HPLC) analysis for the purpose of batch to batch quality control using an Agilent 1260 Infinity system with quaternary gradient pumps, inline degasser, autosampler, column heater, diode array detector and acquisition system (OpenLab CDS ChemStation, Agilent Technologies, Santa Clara, CA, USA). An Agilent Zorbax Eclipse XDB-C18 Analytical 4.6×250 mm, 5 micron column was used at a temperature of 40°C. The extract was dissolved in 5% DMSO in H_2_O and a 20 µg injection was eluted at a flow rate of 1 ml/min using a gradient of 2 solvent systems: (A) 0.1% formic acid in H_2_O; (B): 0.1% formic acid in ACN. The mobile phase was 98% A at time 0, 88% at 34 min. with a 16 min. hold, 75% at 70 min. 5% at 82 min. with a 16 min. hold, followed by a hold at 98% for 15 min. Chromatograms were compared with those of the original samples to ensure the presence of major peaks prior to use in bioassays [41]. Quality tested batches of 220D-F2 were suspended in DMSO (stock of 20 mg/ml or 50 mg/ml), sterile filtered (0.2 µm), and stored in sterile vials at −80°C prior to use in all bioassays.

### Preparation of Inocula to Challenge Planktonic Cells and for Biofilm Assays

An overnight BAP culture of *S. pneumoniae* strains was used to prepare a cell suspension in THY broth to an optical density at 600 nm (OD_600_) of 0.05 and incubated at 37°C in 5% CO_2_ atmosphere until the culture reached an OD_600_ of 0.2 (early log phase). An aliquot (∼7×10^5^ CFU/ml) was inoculated in duplicate into either an 8-well glass slide (Lab-Tek, Rochester, NY) or a polystyrene 24-well microtiter plate (Costar, Corning, NY) containing THY broth and incubated at 37°C with 5% CO_2_ for the indicated time.

### Antimicrobial Effects of 220D-F2 Against Planktonic Cells

To study the effect of 220D-F2 on planktonic cells, strains were inoculated in THY broth, in 24-well polystyrene microtiter plates, and immediately treated with either DMSO or two different concentrations of 220D-F2 (100 µg/ml and 200 µg/ml). Treated bacteria were statically incubated at 37°C with 5% CO_2_ atmosphere for 3 h and CFU/ml of planktonic cells or planktonic cells-derivative biofilms were obtained as follows: planktonic cells were gently removed, serially diluted in phosphate buffered saline (PBS, pH = 7.4) and plated onto BAP to obtain CFU/ml. Once supernatant-containing planktonic cells were completely removed, biofilms were gently washed with phosphate buffered saline (PBS) to eliminate unbound bacteria, 1 ml of sterile PBS was added and then biofilms were scraped thoroughly, including well edges. Biofilm suspensions were serially diluted in PBS and plated on BAP to obtain biofilm viable counts (CFU/ml). Colonies were counted using the Bantex 920A colony counter (American Bantex Corporation, Burlingame, CA).

### Evaluation of Viability of Planktonic Cells by a Fluorescence-based Method

A LIVE/DEAD BacLight bacterial viability kit L7012 (Invitrogen-Molecular Probes) was used to further visualize the viability of planktonic cells challenged with different concentrations of 220D-F2. Fluorescent dyes included in the kit incorporate, or not, into bacterial cells as a function of membrane integrity, and therefore viability. Staining procedure and concentrations of dyes were utilized as per manufacturer’s recommendations. Preparations were observed and photographed utilizing an inverted Evos fl microscope (Advanced Microscopy Group).

### Antimicrobial Effects of 220D-F2 against Preformed Pneumococcal Biofilms

To study the antibacterial effect of 220D-F2 on early and mature biofilms (preformed biofilms), strains were inoculated in THY broth and incubated for 3 or 8 h. The culture medium was then completely removed and biofilms were added with fresh THY broth. These preformed biofilms were treated with DMSO or 220D-F2 (100 µg/ml or 200 µg/ml) and incubated for an additional 3, 6 or 12 h at 37°C with 5% CO_2_ atmosphere. Biofilm counts were then obtained as described above. The minimum biofilm-inhibiting concentration (MBIC) was calculated as the concentration of the extract in which biofilms were eradicated (i.e. killed and/or dispersed) to a level ≥90% for MBIC_90_ or ≥50% for MBIC_50_ in comparison to control wells only treated with DMSO.

### Fluorescence Images of Pneumococcal Biofilms Treated with 220D-F2

To obtain fluorescence images of biofilms treated with the extract 220D-F2, SPJV01 (expressing *gfp* under regulation of the maltose promoter) [50] was inoculated in THY broth supplemented with 2% maltose and immediately treated with either DMSO or different concentrations of 220D-F2. These cultures were statically incubated at 37°C with 5% CO_2_ atmosphere for 8 h. Planktonic cells were then removed and biofilm cells were washed with PBS and fixed with 4% paraformaldehyde overnight. The cells were blocked with 2% Bovine Serum Albumin (BSA) for 1 h and stained for 1 h at room temperature in the dark with a polyclonal anti-*S. pneumoniae* antibody coupled to fluorescein isothiocyanate (FITC; ViroStat, Portland, ME). Preparations were washed once with PBS, mounted with Vectashield mounting medium (Vector Laboratories, Burlingame, CA) and analyzed with a Zeiss LSM 510 confocal microscope. Confocal images were analyzed with LSM Image Browser, version 4.02.121. In another set of experiments, preformed biofilms (8 h) were treated as above for 3, 6 or 12 h. Since GFP production is quenched 8–10 h post-inoculation [47], biofilm preparations were stained with DAPI (100 nM) [4′,6-diamidino-2-phenylindole] for 5 min at room temperature and images photographed using an inverted Evos fl microscope (Advanced Microscopy Group).

### Cell Cultures

Human-derived pharyngeal Detroit 562 cells (ATCC CCL-198) were regularly cultured in Minimum Essential Medium (MEM 1X) (Gibco, Grand Island, NY) supplemented with non-heat inactivated 10% fetal bovine serum (FBS) (Atlanta Biologicals, Flowery Branch, GA), 1% nonessential amino acids (Sigma, St. Louis, MO), 1% glutamine (Sigma, St. Louis, MO), penicillin (100 U/ml) and streptomycin (100 µg/ml). Cells were normally harvested with 0.25% trypsin (Gibco, Grand Island, NY), resuspended in the cell culture medium, and incubated at 37°C in a 5% CO_2_ humidified atmosphere.

### The Bioreactor (biBio) with Living Cultures on Human Nasopharyngeal Cells

Preparation of cell cultures for experiments in the bioreactor (hereafter refered as biBio) was done essentially as recently described [51], [52]. Human pharyngeal cells installed in the biBio were inoculated with an aliquot containing ∼7×10^5^ CFU/ml of *S. pneumoniae* D39 strain and immediately perfused with sterile MEM medium, with a low flow rate (0.20 ml/min), supplemented with non-heat inactivated 10% fetal bovine serum and buffered with 1% HEPES (10 mM) (Gibco, Grand Island, NY). Biofilms were allowed to form for 8 h and treated with DMSO or different concentrations of 220D-F2 (200, 400 or 800 µg/ml) for an additional 12 h period. Higher doses of 220D-F2 were required to kill pneumococci in the biBio. Biofilms were then collected in sterile PBS. The supernatant coming off the apical side of the biBio, and containing planktonic cells, was also collected after treatment with 220D-F2. Both biofilms and planktonic cells were serially diluted in PBS and plated onto BAP to obtain viable counts (CFU/ml). Each concentration was tested in duplicate (technical replicates) and the experiments were conducted on two different days.

### Quantitative Minimum Inhibitory Concentration (qMIC)

A modified quantitative MIC (qMIC) approach, was utilized because the physical aspect (i.e. color, texture, etc.) of the 220D-F2 interfered with classic MIC readings. qMIC was based on recommendations of the Clinical and Laboratory Standards Institute (CLSI) broth microdilution guidelines [53]. Briefly, an overnight culture of the strains was utilized to prepare a suspension in physiological saline which was adjusted to the 0.5 McFarland turbidity standard. An aliquot (100 µl) of this bacterial suspension was added to 11 ml of THY broth, or Cation Adjusted Muller-Hinton broth (CAMHB) with 2–5% lysed horse blood (LHB) as per CLSI guidelines, and used it to serially dilute the extract 220D-F2 in a 96-well plate (Costar, Corning, NY) to obtain a final concentration of 20, 40, 80, 160, or 320 µg/ml. Negative control (THY or CAMHB with LHB), experimental control (bacterial suspension with DMSO) and positive control (bacterial suspension) were included. Treated bacterial suspensions were incubated at 37°C with 5% CO_2_ atmosphere for THY or 35°C at ambient air for CAMHB with LHB for 20 to 24 hours.

To quantify the number of viable pneumococcal cells post-incubation with 220D-F2, we utilized a molecular approach by using quantitative (q)PCR reactions as follows: DNA was extracted from 100 µl of bacterial suspension treated with 100 µl of TE buffer containing 0.04 g/ml of lysozyme and 75 U/ml of mutanolysin, using the automated BioMerieux NucliSENS easyMAG 2.0.1 instrument (BioMerieux, Durham, NC). Extracted DNA (1 µl) was utilized as template in qPCR reactions (25 µl) targeting the *lyt*A gene [54]. These reactions contained 1X Invitrogen Platinum Quantitative PCR SuperMix-UDG, 200 nm of each primer (Forward-5′-ACGCAATCTAGCAGATGAAGCA–3′ and Reverse-5′-TCGTGCGTTTTAATTCCAGCT-3′) and 200 nm of the probe (5′-FAM – GCCGAAAACGCTTGATACAGGGAG –3′ –BHQ1), and molecular biology grade water. Duplicate reactions were run in a CFX96 real-time system thermal cycler (Bio-Rad, Hercules, CA) with the following cycling parameters, initial denaturation at 95°C for 2 min, followed by 40 cycles of denaturation at 95°C for 15 sec and annealing and extension at 60°C for 1 min. To obtain molecular CFU/ml, purified genomic DNA from *S. pneumoniae* reference strain TIGR4 was serially diluted to prepare standards representing 2.14×10^1^, 4.29×10^1^, 4.29×10^2^, 4.29×10^3^, 4.29×10^4^, or 4.29×10^5^ genome copies. A standard curve was constructed and CFU/ml (D39 [44] and TIGR4 [45] encode only one copy of the *lyt*A gene) were calculated using the Bio-Rad CFX manager software. qMIC’s experiments were repeated three times on different days. Efficiency of these qPCR studies was always between ∼95–99%.

### Quantitative MIC by Dilution and Plating

Reference strain D39 and multi-drug resistant strain GA 58771 were chosen for quantitative MIC studies by dilution and plating. Strains were essentially prepared and treated with different concentrations of 220D-F2 as indicated above. At the end of the incubation period, bacteria were serially diluted and plated on BAP to obtain viable counts (CFU/ml). Experiments included technical replicates and were conducted at least three times.

### Extraction of DNA from *S. pneumoniae* Strains

Strains were grown overnight on blood agar plates. Bacterial suspensions were made with 200 µl of sterile PBS and DNA extracted from washed pellets using the QIAamp Mini kit (Qiagen), following the manufacturer’s instructions. Final elution was done in 100 µl of sterile DNA grade water and DNA preparations were stored at −80°C until used.

### Statistical Analysis

The statistical analyses were performed using the Microsoft Excel software (Microsoft corporation). Differences between mean values were calculated by Student’s *t*-test, with *p* value less than 0.05 considered statistically significant.

## Results

### 220D-F2 Inhibits Formation of Pneumococcal Biofilms

**Figure 1:**
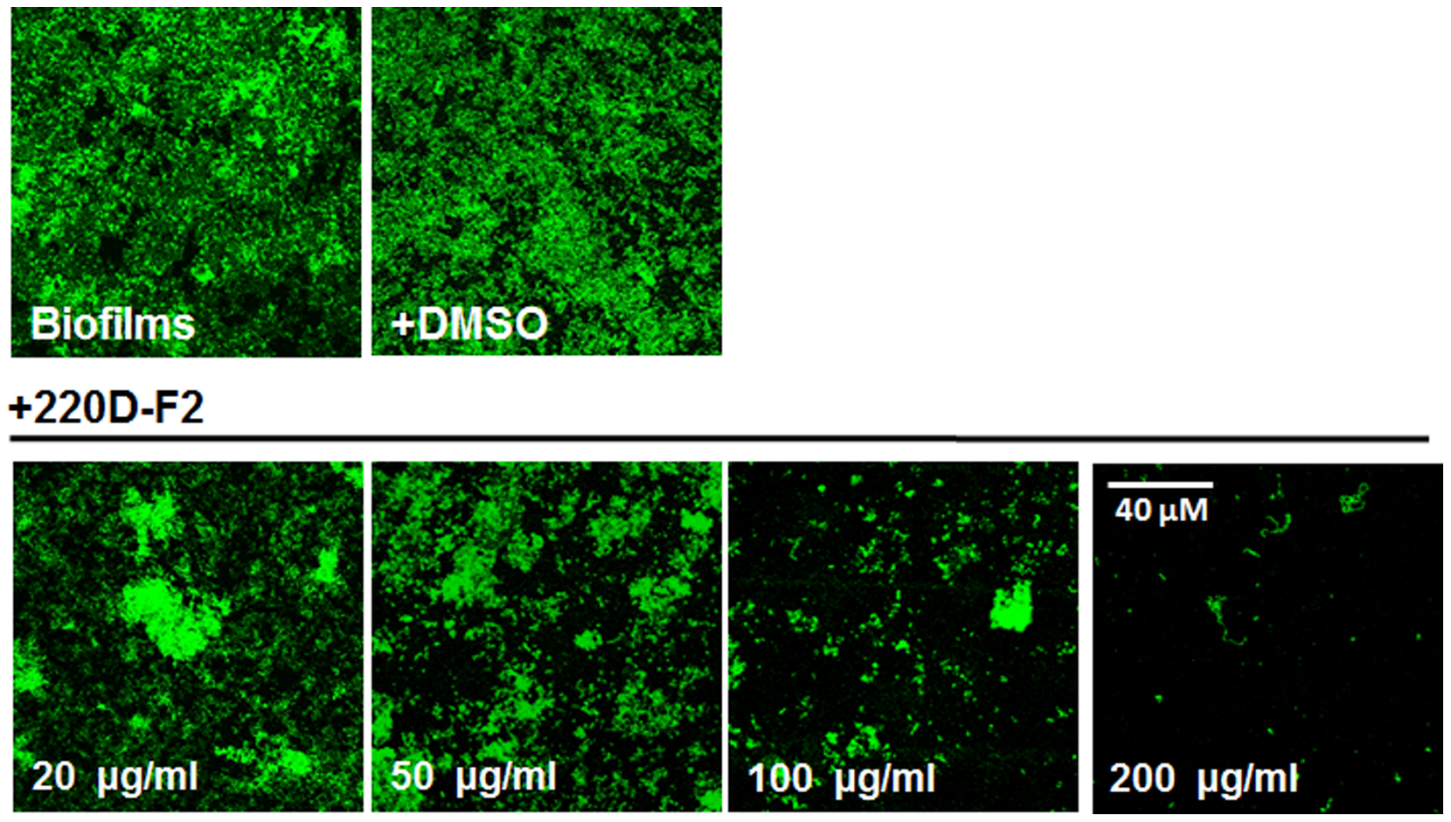
Inhibition of pneumococcal biofilms by 220D-F2. SPJV01 was inoculated in 96 well-plates containing THY and treated with DMSO or the indicated concentration of 220D-F2. Treated cultures were incubated for 8 h at 37°C. Micrographs of fluorescent biofilms were obtained by confocal microscopy. Scale bar shown at the bottom left panel is valid for all panels. Shown is a representative of five independent experiments.

We and others have previously demonstrated that biofilms made by invasive *S. pneumoniae* strain D39 are completely formed between 8 to 10 h post-inoculation. We also demonstrate that D39, and SPJV01 a D39 isogenic derivative, produces more biofilm biomass than strains TIGR4 or strain R6 [47]. To begin exploring the inhibitory effects that 220D-F2 would have on these structures, we inoculated SPJV01 with increasing amounts of 220D-F2, and treated bacteria were incubated for 8 h. As this extract was dissolved in DMSO, for the control wells included along with strain D39 the same volume of DMSO (µl) was utilized as for each of the tested concentrations of 220D-F2. Our results demonstrate dose-dependent inhibition of formation of pneumococcal biofilms treated with 220D-F2 but DMSO did not have an apparent inhibitory effect on the formation of biofilms (not shown). Confocal microscopy micrographs of biofilms formed, when incubated with DMSO, show normal structure (i.e. dense zones of bacterial aggregates) whereas those structures formed when in the presence of 220D-F2 show a dose-dependent decrease of attached bacteria (Fig. 1).

### 220D-F2 Inhibits Formation of Pneumococcal Biofilms by Killing Planktonic Cells

**Figure 2:**
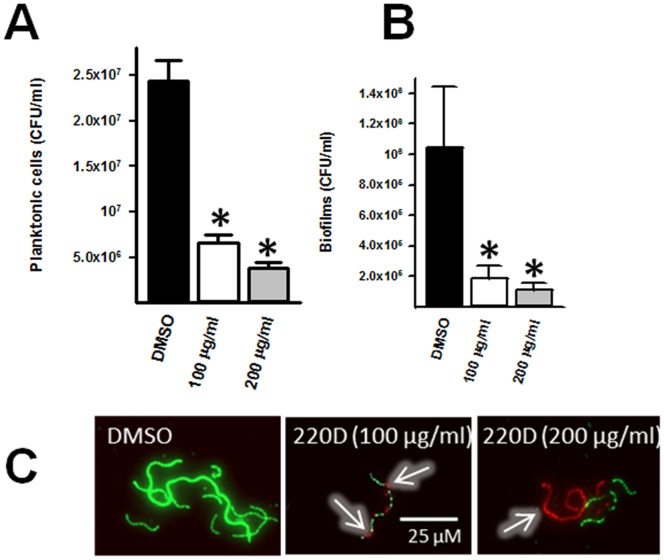
Killing of planktonic pneumococci by 220D-F2. *S. pneumoniae* strain D39 was inoculated in 24 well-plates containing THY and treated with DMSO or the indicated concentration of 220D-F2; treated cultures were incubated for 3 h at 37°C. Planktonic cells were removed (A) and then biofilms were washed and removed (B). Both populations were diluted and plated onto BAP to obtain CFU/ml. Error bars represent the standard error of the mean calculated using data from two independent experiments processed in duplicate. Statistical significance (*p*≤0.05) was calculated using a non-parametric one-tailed Student’s *t*-test (*). C) Planktonic pneumococci treated for 3 h were also stained by the LIVE/DEAD assay and imaged using a fluorescent microscope. Panels show the merge of the green and red channel. White arrows points out dead pneumococci. Scale bar is valid for all panels.

Since the above experiment inoculated 220D-F2 and planktonic cells at the same time, to investigate whether inhibition of biofilm formation was due to the killing of planktonic cells, viability of planktonic cultures was evaluated by dilution and plating. In comparison to DMSO-treated control, planktonic cultures treated with either 100 µg/ml or 200 µg/ml of 220D-F2 showed a significant reduction of bacterial viability (Fig. 2A). Fluorescence micrographs of planktonic pneumococci treated with 220D-F2 and stained with the LIVE/DEAD assay confirmed the presence of pneumococci with disrupted membranes (Fig. 2C). As expected, as there was a reduction in planktonic cells, our studies demonstrate that biofilm counts were also reduced compared to biofilm counts in DMSO-treated control wells (Fig. 2B). Together, these results indicate that 220D-F2 kills planktonic cultures of *S. pneumoniae* strain D39, as early as 3 h post-inoculation, thereby reducing the population of pneumococci that forms the biofilm structure.

### Inhibitory Effect of Extract 220D-F2 against Biofilms

We next evaluated the potential of 220D-F2 as an inhibitor against early and mature pneumococcal biofilms made by strain D39 [47].

**Figure 3:**
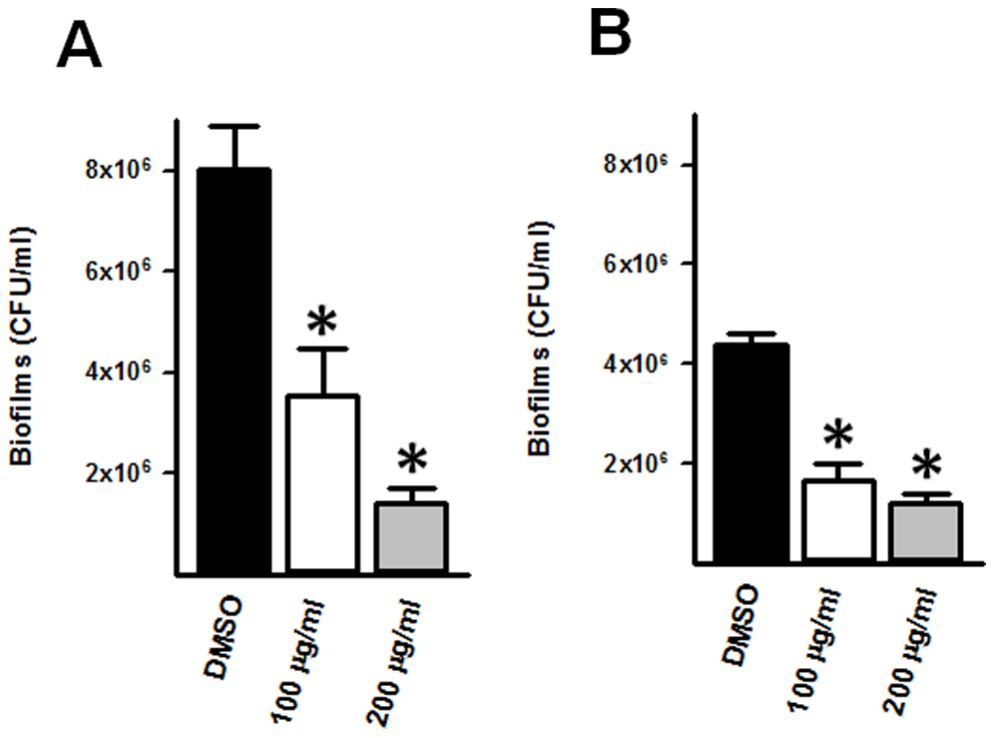
Killing of early pneumococcal biofilms by 220D-F2. *S. pneumoniae* D39 was incubated for 3 h at 37°C after which early biofilms were washed and added with fresh THY containing the indicated concentration of 220D-F2 or DMSO. Treated biofilms were incubated for (A) 3 or (B) 6 h at 37°C and then washed, diluted and plated onto BAP to obtain CFU/ml. Error bars represent the standard error of the mean calculated using data from two independent experiments; each experiment was processed in duplicate. Statistical significance (*p*≤0.05) was calculated using a non-parametric one-tailed Student’s *t*-test (*).

**Figure 4:**
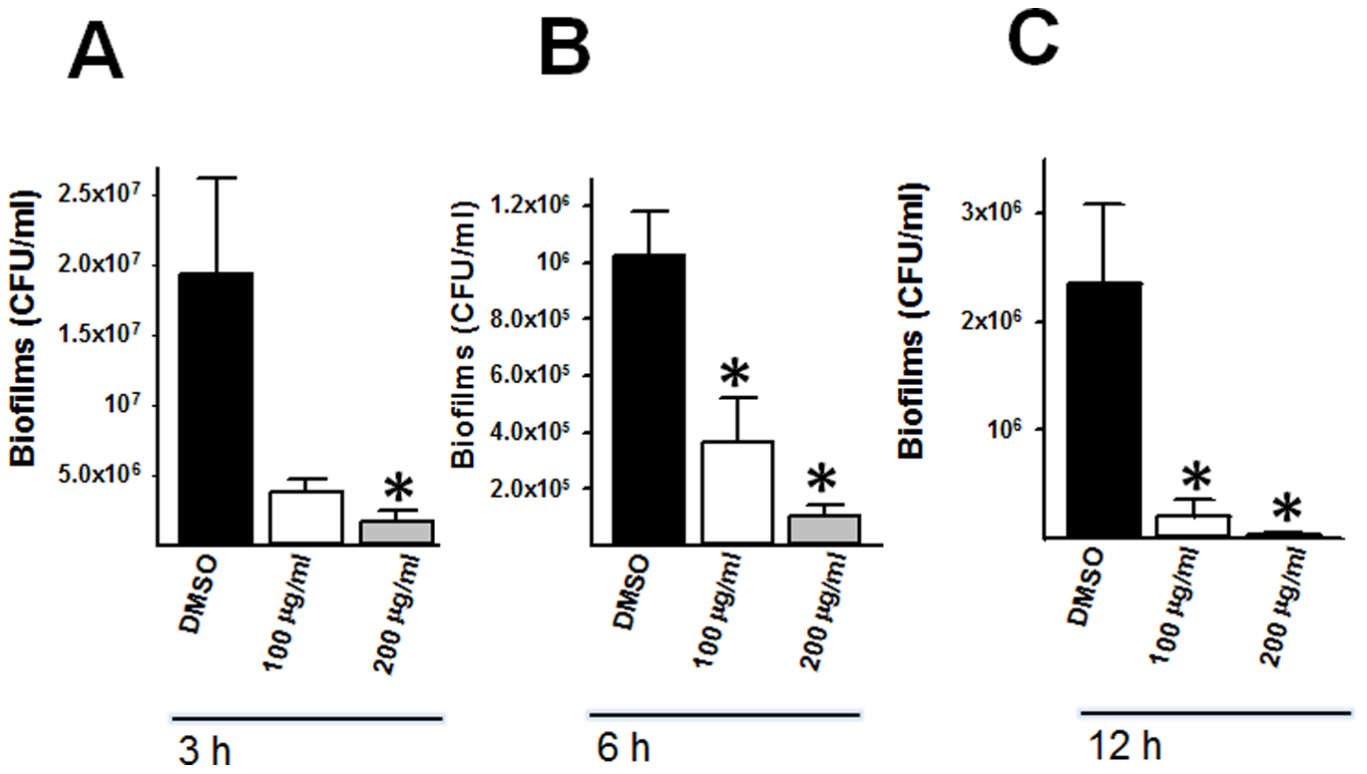
Killing of mature pneumococcal biofilms by 220D-F2. *S. pneumoniae* D39 was inoculated and incubated for 8 h at 37°C after which mature biofilms were washed and added with fresh THY containing the indicated concentration of 220D-F2 or DMSO. Treated biofilms were incubated for (A) 3, (B) 6, or (C) 12 h at 37°C and then washed, diluted and plated onto BAP to obtain CFU/ml. Error bars represent the standard error of the mean calculated using data from two independent experiments; each experiment was processed in duplicate. Statistical significance (*p*≤0.05) was calculated using a non-parametric one-tailed Student’s *t*-test (*).

Early biofilm structures were treated with increasing amounts of the extract 220D-F2 and then incubated for either 3 or 6 h. We also treated mature biofilm structures with 220D-F2 for an additional 12 h period. Preformed biofilms produced 3 h post-inoculation and then additionally treated with 220D-F2 for 3 h showed a significant reduction of the biofilm biomass (Fig. 3A). Similarly, preformed biofilms produced 3 h post-inoculation and then treated for an additional 6 h showed significant reduction of biofilms (Fig. 3B). Biofilm counts were similarly obtained whether 100 µg/ml or 200 µg/ml of 220D-F2 was utilized. The calculated minimum biofilm inhibitory concentration (MBIC) revealed that treatment of early biofilms for 3 or 6 h with 100 or 200 µg/ml of 220D-F2 obtained MBIC_50_. Mature biofilms were similarly challenged with 100 and 200 µg/ml of 220D-F2. Consistent with a dose-dependent effect, mature pneumococcal biofilms treated for 3 h with 100 µg/ml of 220D-F2 did not show a significant decrease of biofilm cells (Fig. 4A) whereas treatment for 6 or 12 h with the same amount induced a significant decrease of biofilm counts (Figs. 4B and 4C). The calculated MBIC of preformed mature biofilms treated for 3, 6 or 12 h with 100 and 200 µg/ml of 220D-F2 resulted in a MBIC_50_ or MBIC_90_, respectively. Altogether, our results demonstrate that 220D-F2 induces a significant reduction in preformed biofilm cells.

**Figure 5:**
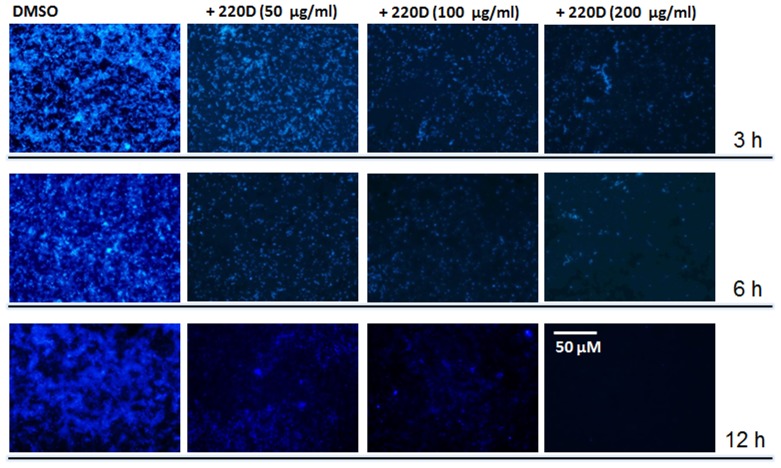
Micrographs of 220D-F2 incubated with mature pneumococcal biofilms. *S. pneumoniae* D39 was inoculated and incubated for 8 h at 37°C after which biofilms were washed and added with fresh THY containing the indicated concentration of 220D-F2 or DMSO. These treated mature biofilms were incubated for 3, 6 or 12 h and after washes, the biofilm structure was stained with DAPI (100 nM). Stained biofilms were imaged by fluorescence. Scale bar shown is also valid for all panels.

To further explore whether the 220D-F2-treated biofilm structure had been dispersed, i.e. detached from the substrate, biofilms were stained by fluorescence. Fluorescence micrographs of mature biofilms treated for 3, 6 or 12 h with 50 µg/ml of 220D-F2 revealed moderate detachment of the biofilm structure. However, when incubated with 100 or 200 µg/ml for 12 h, biofilms had been almost completely detached from the substrate (Fig. 5).

### 220D-F2 Kills Pneumococcal Biofilms and Planktonic Cells Formed on Human Pharyngeal Cells

To assess whether 220D-F2 would kill *S. pneumoniae* growing in a more natural environment, strain D39 was inoculated in the biBio and incubated for 8 h. The biBio simulates the nasopharyngeal environment as it has human pharyngeal cells and a continuous flow of nutrients. *S. pneumoniae* in the biBio was then incubated with different amounts of 220D-F2 and the viability of biofilms and planktonic cells was evaluated by dilution and plating. Whereas 200 or 400 µg/ml did not change the biofilm biomass (Table 1), incubating the biBio with 800 µg/ml of 220D-F2 for 12 h significantly reduced pneumococcal biofilms (MBIC_50_) (Table 1). At the same concentration counts of planktonic cells were also significantly reduced (MBIC_90_).

**Table 1:** Treatment of *S. pneumoniae* D39 grown in the biBio on human pharyngeal cells.

*S. pneumoniae*	CFU/ml
**Planktonic cells**	
DMSO	5.72×10^5^±3.70×10^5^
220D-F2:	
200 µg/ml	2.02×10^6^±8.43×10^5^
400 µg/ml	5.23×10^6^±2.57×10^6^
800 µg/ml*	1.10×10^4^±9.80×10^3^
**Biofilms**	
DMSO	3.54×10^7^±1.53×10^7^
220D-F2:	
200 µg/ml	2.63×10^8^±7.24×10^7^
400 µg/ml	1.12×10^8^±3.25×10^7^
800 µg/ml*	1.08×10^7^±3.93×10^6^
±standard error	

*p<0.05 in comparison to DMSO.

### Molecular Studies of Minimum Inhibitory Concentration (MIC)

Our results indicate that 220D-F2 may have potential therapeutic applications against pneumococcal diseases. Therefore, we next investigated the minimum inhibitory concentration of 220D-F2 utilizing a quantitative (q)MIC protocol. This modified protocol included molecular quantification, and bacterial counts, of *S. pneumoniae* D39 cells (CFU/ml) after exposure to those tested 220D-F2 dilutions. Our molecular approach utilized a quantitative PCR assay targeting the autolysin gene *lyt*A [54]. Planktonic cells were diluted to the recommended cell density and incubated with the vehicle (DMSO) or serial dilutions of 220D-F2 starting at 20 µg/ml. In comparison to the untreated control, treatment with DMSO, 20 or 40 µg/ml of 220F-D2 resulted in a similar bacterial density (Table 2) whether incubated in THY or CAMHB with LHB. In contrast, treatment with 80 µg/ml (Table 2) reduced bacterial viability by four orders of magnitude whereas 160, or 320 µg/ml reduced pneumococcal cells down to the lower limit of detection of our assay (<100 CFU/ml) indicating that 220D-F2 had killed almost, if not all, pneumococci. Similar treatment of reference strain TIGR4 or three other antibiotic resistant strains resulted in similar eradication of pneumococci (Table 2). Dilution and plating of pneumococcal strains treated with different concentrations of 220D-F2 showed similar bacterial counts as those obtained using our molecular approach (Table 3).

**Table 2:** Bactericidal activity of 220D-F2 against *S. pneumoniae* strains.

Strain/Treatment	CFU/ml
**D39**	
Untreated	5.48×10^8^±3.91×10^6^
DMSO	4.33×10^8^±2.91×10^7^
220D-F2:	
20 µg/ml	1.61×10^8^±2.18×10^7^
40 µg/ml	3.41×10^8^±5.91×10^7^
80 µg/ml	2.70×10^4^±1.40×10^4^
160 µg/ml	≤100±0.00
320 µg/ml	≤100±0.00
**TIGR4**	
Untreated	1.43×10^7^±2.59×10^6^
220D-F2 (80 µg/ml)	≤100±0.00
**TN33388** *	
Untreated	9.58×10^7^±8.55×10^6^
220D-F2 (80 µg/ml)	≤100±0.00
**7828-04** *	
Untreated	1.57×10^8^±3.66×10^7^
220D-F2 (80 µg/ml)	≤100±0.00
**GA58771** *	
Untreated	8.73×10^7^±1.13×10^7^
220D-F2 (80 µg/ml)	≤100±0.00

±standard error,

*antibiotic resistant strain.

Experiments were repeated three times.

**Table 3:** Bactericidal activity of 220D-F2 against *S. pneumoniae* strains quantified by culture.

Strain/treatment	CFU/mL
D39	
Untreated	3.54×10^8^±1.49×10^8^
DMSO	1.00×10^8^±7.61×10^7^
220D-F2 (80 µg/ml)	<100±0.00
GA58771*	
Untreated	6.82×10^7^±2.95×10^7^
DMSO	1.97×10^7^±1.37×10^7^
220D-F2 (80 µg/ml)	<100±0.00

±standard error,

*antibiotic resistant strain.

Experiments were repeated three times.

## Discussion

To the best of our knowledge, this is the first report of an agent with antibacterial activity for *S. pneumoniae* planktonic cells, and pneumococcal biofilms, obtained from a botanical product. 220D-F2 was able to kill preformed pneumococcal biofilms reducing the population of biofilm cells 6 or 12 h post-treatment with 200 µg/ml, to ∼10% or ∼1%, respectively.

A clear dose response effect allowing killing and detachment of preformed pneumococcal biofilms was observed. Complete detachment of the biofilm structure from the substratum was observed after 12 h of treatment with 200 µg/ml (Fig. 5), which correlated well with bacterial counts showing a reduction of ∼99% of the biofilm biomass (Fig. 4C). Moreover, using a biofilm model with human pharyngeal cells, treatment of pneumococcal biofilms with 800 µg/ml of 220D-F2 induced a significant reduction of the biofilm biomass (Table 1). The observation that a higher amount of 220D-F2 was required to kill *S. pneumoniae* biofilms in the biBio with human pharyngeal cells (800 µg/ml) vs the static model (200 µg/ml) might be due to a combination of different factors. For example, the biBio is constantly perfused, at a fixed rate (200 µl/min), with cell culture medium containing 220D-F2 that might delay the exposure time of this compound’s active molecules with pneumococci [3]. Unknown metabolites produced by living cultures of human pharyngeal cells exposed to pneumococci may also cause indirect inhibition of 220D-F2. There is also an increased biofilm biomass when those pneumococcal biofilms are grown on human pharyngeal cells that may account for the increased concentration of 220D-F2 required to kill pneumococci in this *in vivo* model [40], [51].

Only a few other molecules have proven effective against pneumococcal biofilms. A recent study by Domenech et al (2011) found a ∼80% reduction of pneumococcal biofilms when preformed biofilms were incubated with the amidase LytA, which is encoded and produced by the pneumococcus and other related streptococci [55]. Dispersion of ∼70% of pneumococcal biofilms have been also achieved incubating with 5-azacytidine (5-aza), an analog of the pyrimidine nucleoside cytidine [56]. A paper by Trapetti et al (2009) showed that neuraminidase inhibitors DANA (i.e., 2,3-didehydro-2-deoxy-N-acetylneuraminic acid), zanamivir, and oseltamivir inhibit the capacity of pneumococci to form sialic acid-dependent biofilms [57]. Whereas activity of 5-aza against other pathogens has not been investigated, LytA and neuraminidase inhibitors appear to be specific to pneumococcal biofilms. Studies within this work added a botanical derivative extract to this list. Additional advantages of 220D-F2 include that it is also active against biofilms made by antibiotic resistant, MRSA strains, and does not induce apparent damage to a variety of *in vitro* cultured eukaryotic cells [i.e. human kidney proximal tubular (HK-2) cells, rat kidney (NRK-52E) cells, mouse kidney proximal tubular cells, and mouse hepatocytes (AML12)] [41].

Detachment of biofilms was mainly mediated by reduction of bacterial viability as the LIVE/DEAD assay confirmed disrupted membranes (Fig. 2C). Planktonic pneumococci were almost completely eradicated by 220D-F2 showing a MIC of 80 µg/ml. These strains included multidrug resistant pneumococci as well as another reference strain TIGR4. Unlike other natural products that have been tested against planktonic pneumococci and *S. aureus*, [58]–[60] we demonstrate in this work that active molecules within 220D-F2 bear potential to treat both biofilm-related diseases and those pneumococcal diseases mediated by planktonic organisms [i.e. pneumococci growing in liquid microenvironments such as in blood or cerebrospinal fluid (CSF)].

220D-F2 contains a mixture of ellagic acid and ellagic acid derivatives and other minor constituents that remain to be determined [41]. Whereas some of 220D-F2 bactericidal properties may be attributed to ellagic acid and its glycosidic derivatives, the other constituents may also play a significant role in killing planktonic pneumococci and pneumococcal biofilms. Ongoing studies are focused on the fractionation and isolation of individual constituents found in 220D-F2 for structural elucidation and activity testing. We hypothesize that MICs and MBICs of the individual constituents will be lower than those observed in this study for 220D-F2.

This work also introduced the use of molecular reactions, along with MICs, to quantify bacterial density post antimicrobial challenge. We took advantage of quantitative reactions that have been used by our group and others to molecularly quantify pneumococcal load and therefore have quantitative, rather than qualitative, MICs. Bacterial counts obtained using these reactions correlated with those CFU/ml obtained by dilution and plating. Data presented in Table 2 and 3 showed a dramatic drop when planktonic pneumococci were incubated with 80 µg/ml. Therefore, ∼10^5^ CFU/ml of planktonic pneumococci, as inoculated per CLSI guidelines, were eradicated with 80 µg/ml of 220D-F2. A similar pneumococcal load has been reported present in lung tissue of pneumonia patients (≥10^4^ CFU/g) or in their secretions (≥10^5^ CFU/ml) [61]. These data support further fractionation of 220D-F2 and testing against the pneumococcus and other pathogens.

The possibility that qPCR inhibitors contained in the extract interfered with our reactions was refuted as another set of control reactions was added, ∼400 pg of pure *S. pneumoniae* DNA to tubes containing pneumococci treated with 80 µg/ml of 220D-F2, and reactions detected and quantified a similar amount of this DNA control.

In conclusion, this work represents the first study to evaluate the bactericidal activity of the extract 220D-F2 against both *S. pneumoniae* planktonic cells and pneumococcal biofilms. The antibacterial potential of individual constituents within this composition for the treatment of pneumococcal disease produced by multidrug resistant strains, such as chronic otitis media, is promising and worth pursuing. As pneumococcal strains resistant to several antibiotics are increasingly emerging, the discovery of new alternatives such as those active molecules present in 220D-F2 may provide therapeutic alternatives for treating pneumococcal diseases in the future.
